# Fracture behavior of root-amputated teeth at different amount of periodontal support – a preliminary in vitro study

**DOI:** 10.1186/s12903-019-0958-3

**Published:** 2019-11-27

**Authors:** Balázs Szabó, Sufyan Garoushi, Gábor Braunitzer, Balázs Szabó P., Zoltán Baráth, Márk Fráter

**Affiliations:** 1 Department of Periodontology Faculty of Dentistry, University of Szeged, Szeged, Hungary; 2 Department of Biomaterials Science and Turku Clinical Biomaterials Center –TCBC Institute of Dentistry, University of Turku, Turku, Finland; 3 dicomLAB Dental Ltd., Szeged, Hungary; 4 Department of Food Engineering Faculty of Engineering, University of Szeged, Szeged, Hungary; 5 Department of Prosthodontics Faculty of Dentistry, University of Szeged, Szeged, Hungary; 6 Department of Operative and Esthetic Dentistry Faculty of Dentistry, University of Szeged, Tisza Lajos Krt., 64-66, Szeged, H-6720 Hungary

**Keywords:** Furcation, Fracture resistance, Overlay, Short fibre-reinforced composite, Bone support

## Abstract

**Background:**

The purpose of this study was to evaluate the effect of the amount of periodontal support on the fracture resistance of root-amputated maxillary molar teeth restored with either direct class. I. restorations or class II. mesio-occluso-distal (MOD) indirect overlay restorations with cuspal coverage.

**Methods:**

Sixty sound maxillary first molars were collected and randomly divided into four groups. In Groups 1 and 2, MOD cavities were prepared and all cusps were reduced by 2 mm, whereas in Group 3 and 4, only a conservative Class I. cavity was prepared. Subsequently, root canal treatment was performed and the mesio-buccal roots were amputated. Groups 1 and 2 were restored with indirect composite overlay, while Groups 3 and 4 received direct composite fillings. After restoration, teeth were embedded as follows: Groups 1 and 3: normal bone level, Groups 2 and 4: furcation involvement. The specimens were submitted to static fracture resistance testing. Fracture thresholds and fracture patterns were measured and evaluated.

**Results:**

Group 1 had the highest fracture resistance (2311,6 N) among the restored groups and showed statistically significant difference compared to Group 2 (*p* = 0.038) and Group 4 (*p* = 0.011). There was no statistically significant difference in terms of fracture resistance between the rest of the groups. In terms of the fracture patterns, Group 3 was characterized by the highest percentage (60%) of mostly favorable fractures, while the rest of the groups showed predominantly unfavorable ones.

**Conclusions:**

The amount of periodontal support seems to influence the fracture resistance of root-amputated and restored maxillary molars.

## Background

The treatment of periodontally involved maxillary molars can be challenging, given the presence of furcations, root proximities, and the closeness of the maxillary sinus [1, 2]. It has been previously shown that among periodontally compromised teeth, maxillary molars are the most likely to be lost [3, 4]. One of the reasons behind this phenomenon could be that maxillary molars have a unique root morphology and when attachment loss extends to the furcation, a number of problems arise [5]. By the time the furcation has been exposed, more than 30% of the available attachment surface has been lost [6]. Furthermore, due to the poor accessibility of the exposed furcal area, molar teeth respond less favorably to non-surgical periodontal treatment than single-rooted teeth [1]. Nevertheless, patients prefer to keep their own dentition, and the advances in dentistry make it possible, so teeth that would once be removed are now conservatively treated [7]. Thus, root amputations, root resections and bisections have become relatively frequent. Root amputation is the surgical procedure by which one or more of the roots of a multirooted tooth are removed at the level of the furcation whilst the crown and remaining roots are left in function [8].

Root amputation can be a valuable procedure when the tooth in question has a high strategic value or when specific problems exist associated with treatment alternatives such as dental implants (e.g.: limited bone due to destruction or due to proximity of the maxillary sinus, periodontally compromised and smoking patients, etc.) [1]. The indications for root amputation can be divided into two categories: periodontal and endodontic. Conventional periodontal indications include: moderate to advanced furcation involvement, severe bone loss affecting one or more root(s), severe recession or dehiscence of a root or unfavorable root proximity between adjacent teeth [9]. Endodontic indications could include: root fracture or perforation, external root resorption, failed root canal treatment, root caries or endodontic–periodontal combined lesions [9]. The factors to be considered when deciding which root to remove are as follows: the amount of supporting tissue around the roots, the root and root canal anatomy in relation to the endodontic treatment and the periapical condition [10, 11]. The amount of supportive tissue around the roots, which is of key importance regarding the stability and prognosis of the treated tooth, can vary based on whether the indication is a periodontal one or an endodontic one.

Also, it is important to emphasize that as soon as root amputation is indicated, endodontic therapy of the remaining root canals becomes necessary and should be completed prior to the surgical intervention. In general, the prognosis of endodontically treated teeth depends not only on the success of endodontic therapy, but also on the type of coronal reconstruction. Previously it was recommended that a root-amputated tooth should be restored with a full coverage crown [12]. With current adhesive restorations it is possible to restore function and reinforce the tooth without having to sacrifice considerable amounts of healthy tooth structure. Frankenberger et al. and Rocca et al. showed that if a Class I. cavity remains after endodontic treatment, the tooth can safely be restored with a direct composite restoration [13, 14]. However, if one or both marginal ridges are missing after endodontic treatment, restoration with cuspal coverage is highly recommended even in non-root-amputated cases [15, 16]. The question arises, whether the remaining bone level will affect the performance of the restoration-tooth complex in a more minimal invasive (Class I. direct) and a more invasive (Class II. MOD indirect) restorative solution in root-amputated maxillary molar teeth. In this study, we asked this question. The null hypotheses were that there would be no difference in the maximal fracture resistance or in fracture pattern between the tested groups.

## Methods

### Sample selection

180 maxillary molars and 80 maxillary premolars extracted for periodontal or orthodontic reasons were selected for this study. Teeth were kept in 0.9% saline solution at room temperature until use all within 2 months of extraction. During the sample preparation process, all soft tissues covering the root surface was removed with hand scalers. The first inclusion criteria were visual absence of caries or root cracks, absence of previous endodontic treatment, posts or crown or resorptions. Selection based upon the coronal dimensions was performed by the parameters of Fráter and colleagues [17, 18]. Teeth with severe polymorphism of the coronal structures were excluded from the investigation. About 80 % of the specimens ranged 10.0 to 10.9 mm in size, measured at the widest bucco-palatal dimension, and the rest were between 11.0 and 12.0 mm. The mesio-distal dimension of the specimens was also measured, and this parameter allowed a maximum deviation of 10% from the determined mean. Also, root length was standardized as follows: mesio-buccal: 12–14 mm, disto-buccal: 11–13 mm, palatal 12–15 mm. Based on these criteria, sixty maxillary first molars were selected. The rest of the molar and premolar teeth were set aside to be used during the embedding procedure as adjacent teeth (see later).

### Cavity preparation and root canal treatment

Teeth were distributed into four groups (*n* = 15). All procedures were performed by the same trained operator. In Group 1 and 2 standardized MOD cavities were prepared according to Cara et al. [19]. The bucco-palatal width (BPW) of the approximal box of each cavity was two-thirds of the BLW of the tooth, and the occlusal isthmus was half the BPW. In addition, the cavity depth at the occlusal isthmus was standardized to 3.5 mm from the tip of the palatal cusp and 1 mm coronal the cemento-enamel junction (CEJ) at the cervical aspect of the approximal boxes. Finally, all cusps were reduced by 2 mm of their original height. After cavity preparation, the roof of the pulp chamber was removed, and root canal treatment was initiated. Teeth in Groups 3 and 4 received a Class I. cavity preparation which was continued into a traditional endodontic access (TEC) following the principles of TECs as previously reported [20, 21].

After cavity preparation, endodontic treatment was performed on each specimen. The root canals were instrumented with Pathfiles (1–2-3) and ProTaper (S1-S2-F1-F2-F3) (Dentsply Maillefer) to the working length. The specimens were irrigated with 5% NaOCl alternated with 10% EDTA (ethylenediaminetetraacetic acid) with a 2-mL syringe and 25-gauge needle. Root canal filling was performed by matched-single-cone obturation with a master cone (F3 gutta-percha, Dentsply-Maillefer) matching the final instrument used for preparation and sealer (AH plus; Dentsply De Trey GmbH, Konstanz, Germany). Following root canal obturation, a base was applied to the pulp chamber in the form of an approx. 2 mm thick resin modified glass-ionomer barrier (Fuji II LC, GC Europe, Leuven, Belgium). As the base lining set, each mesio-buccal (MB) root was sectioned horizontally at the level of the furcation with a fissure diamond bur (881.31.014 FG – Brasseler USA Dental, Savannah, GA). The sectioned surfaces were smoothened to eliminate any remnants below the sectioning level in order to have a cleansable non-retentive surface.

### Coronal restoration

The cavosurface margins were prepared perpendicular to the tooth surface at the end of the preparation. The cavity was rinsed and air-dried with an air/water syringe. All prepared specimens received the same adhesive treatment. The enamel was acid-etched with 37% phosphoric acid for 15 s, rinsed with water and air-dried. The cavity was adhesive-treated with G-aenial Bond (GC Europe, Leuven, Belgium) according to the manufacturer’s instructions. The adhesive layer was light-cured for 40 s with an Optilux 501 halogen light (Kerr, Orange, CA, USA) at a light intensity of 740+/− 36mWcm^2^. In all groups, an approximately 0.5 mm-thick flow composite layer (G-aenial Flo, GC Europe, Leuven, Belgium) was applied on the floor of the cavity. This layer was light-cured for 40 s. After applying the flowable layer, the missing dentine was rebuilt from short fiber-reinforced composite (SFRC, everX Posterior, GC Europe, Leuven, Belgium). The core material was placed in 2 increments according to the anatomy of the dentine, leaving approx. 2 mm occlusally for the final composite direct or indirect restoration as prescribed by the manufacturer. Each increment was light-cured from the occlusal surface for 40 s.

In Groups 3 and 4, the last occlusal layer was composite material (Gradia Direct Posterior A2, GC Europe, Leuven, Belgium) covering the SFRC. Glycerine gel (DeOx Gel, Ultradent Products Inc., Orange, CA, USA) was applied and final polymerization from each side for 40 s with Optilux 501 was performed.

In Groups 1 and 2 indirect composite overlays were fabricated and luted according to the method in a previous pilot study of Szabó et al. [22]. After refining the cavity margins, polyether impression (Permadyne, 3 M ESPE) was taken of each prepared specimen, using a simultaneous mixing technique according to the manufacturer’s instructions. Provisional restorations were fabricated with Fermit N (Ivoclar Vivadent, Schaan, Lichtenstein), adapted to the cavity, and light- polymerized without interim cement placement. The impressions were molded and composite resin overlays (Gradia Lab, GC Europe, Leuven, Belgium) were prepared by the same technician for each prepared molar specimen. Special attention was paid that the thickness of the overlay should be between 2 and 2.2 mm at every point: after the fabrication of the overlay, the thickness was checked with a digital caliper (Mitutoyo Corp., Kawasaki, Japan) at 9 different points, and occlusal reduction was carried out wherever necessary to have the desired thickness.

At the luting appointment the intaglio surface of the composite overlays was sandblasted, rinsed and ultrasonically cleaned (Emag, Valkenswaard, Netherlands) in distilled water in 5 min. They were then silanized (Ceramic Primer, GC Europe, Leuven, Belgium) and heat dried for 1 min at 100 °C (DI500, Coltene, Altstatten, Switzerland) and coated with a thin layer of adhesive resin (Stick Resin, GC Europe, Leuven, Belgium).

**Fig. 1:**
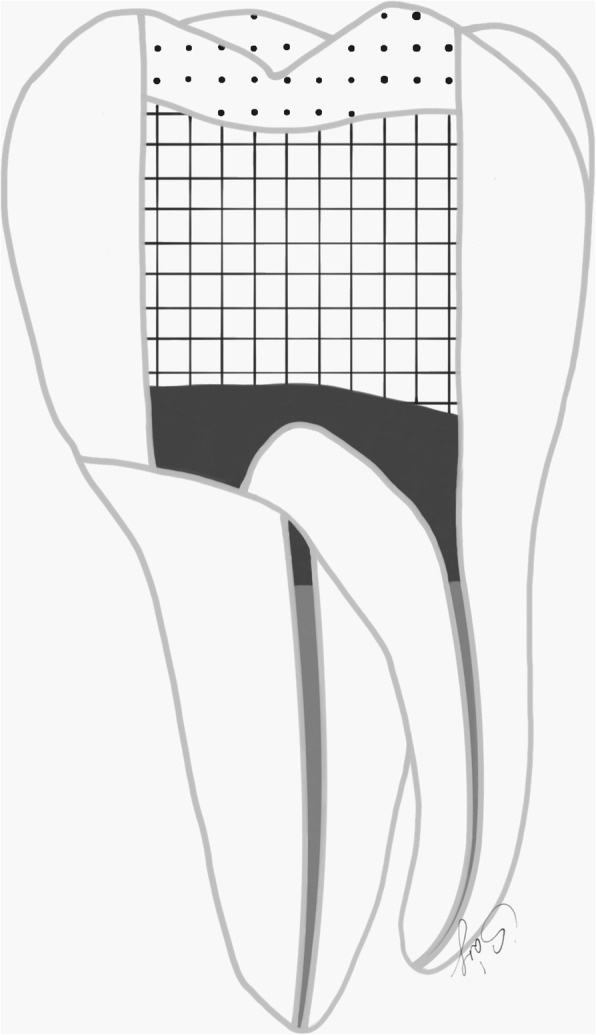
(left) Schematic figure representing the groups (Group 3. and 4.) restored with the direct filling

**Fig. 2:**
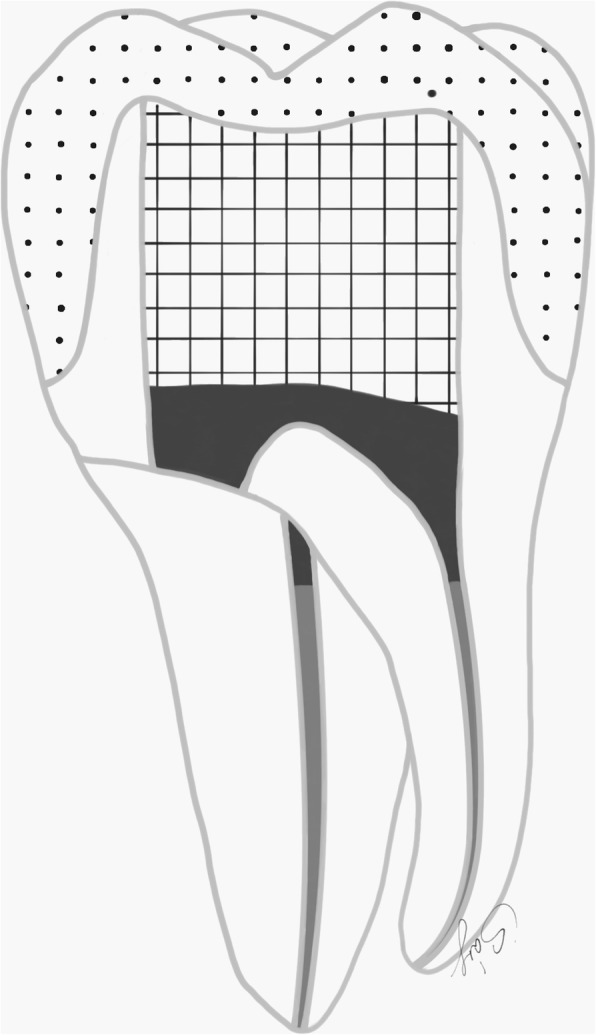
(right) Schematic figure representing the groups (Group 1. and 2.) restored with the indirect overlay

Regarding the prepared tooth, the enamel margins were etched with 37% phosphoric acid for 30 s, rinsed with water and air-dried. Then adhesive resin (Stick Resin, GC Europe, Leuven, Belgium) was applied and air-thinned, but not photopolymerized. The overlays were luted with pre-heated restorative composite resin (Gradia Anterior A2, GC Europe, Leuven, Belgium). The luting agent was applied onto the intaglio surface of the overlays and the overlays were applied on the teeth under finger pressure until complete adaptation. After removing the excess material, glycerine gel (DeOx Gel, Ultradent Products Inc., Orange, CA, USA) was applied and photopolymerization from each side for 40 s with Optilux 501 was performed (Figs. 1 and 2.).

### Embedding the samples

**Fig. 3:**
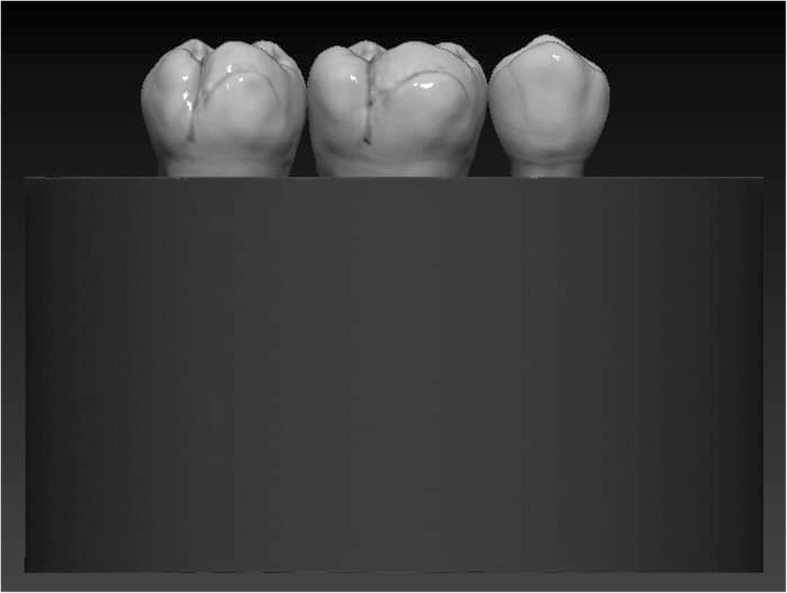
Schematic figure representing the groups (Group 1. and 3.) with a simulated normal bone level

**Fig. 4:**
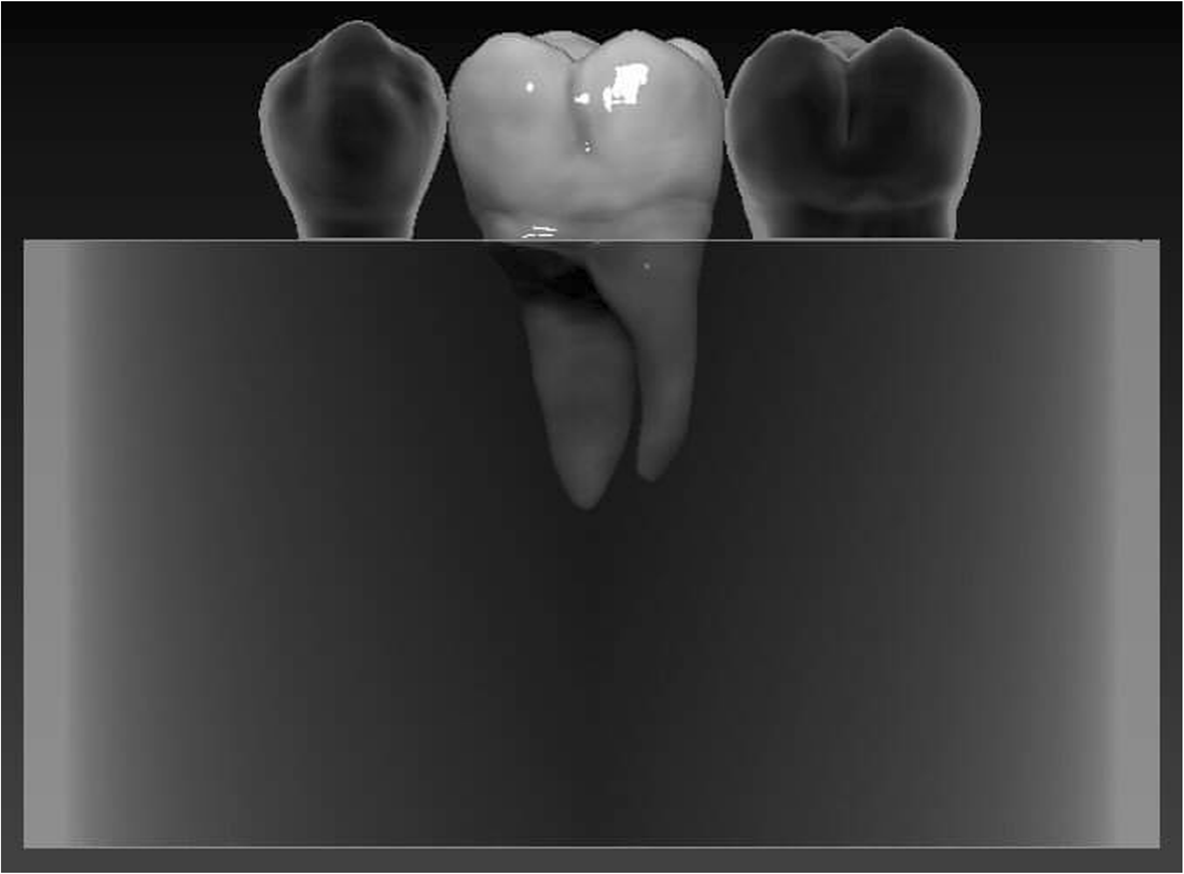
Schematic figure representing the groups (Group 1. and 3.) with a simulated normal bone level

**Fig. 5:**
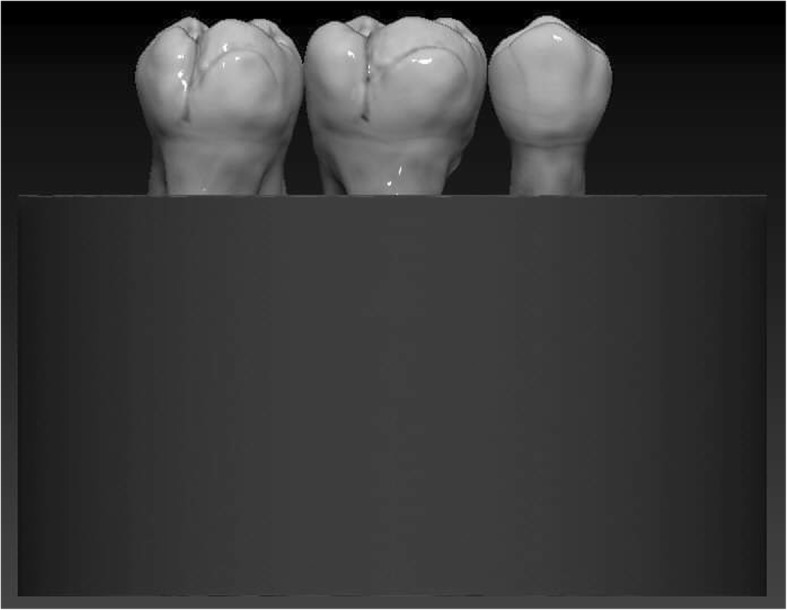
Schematic figure representing the groups (Group 2. and 4.) with a simulated Grade I. furcation involvement

**Fig. 6:**
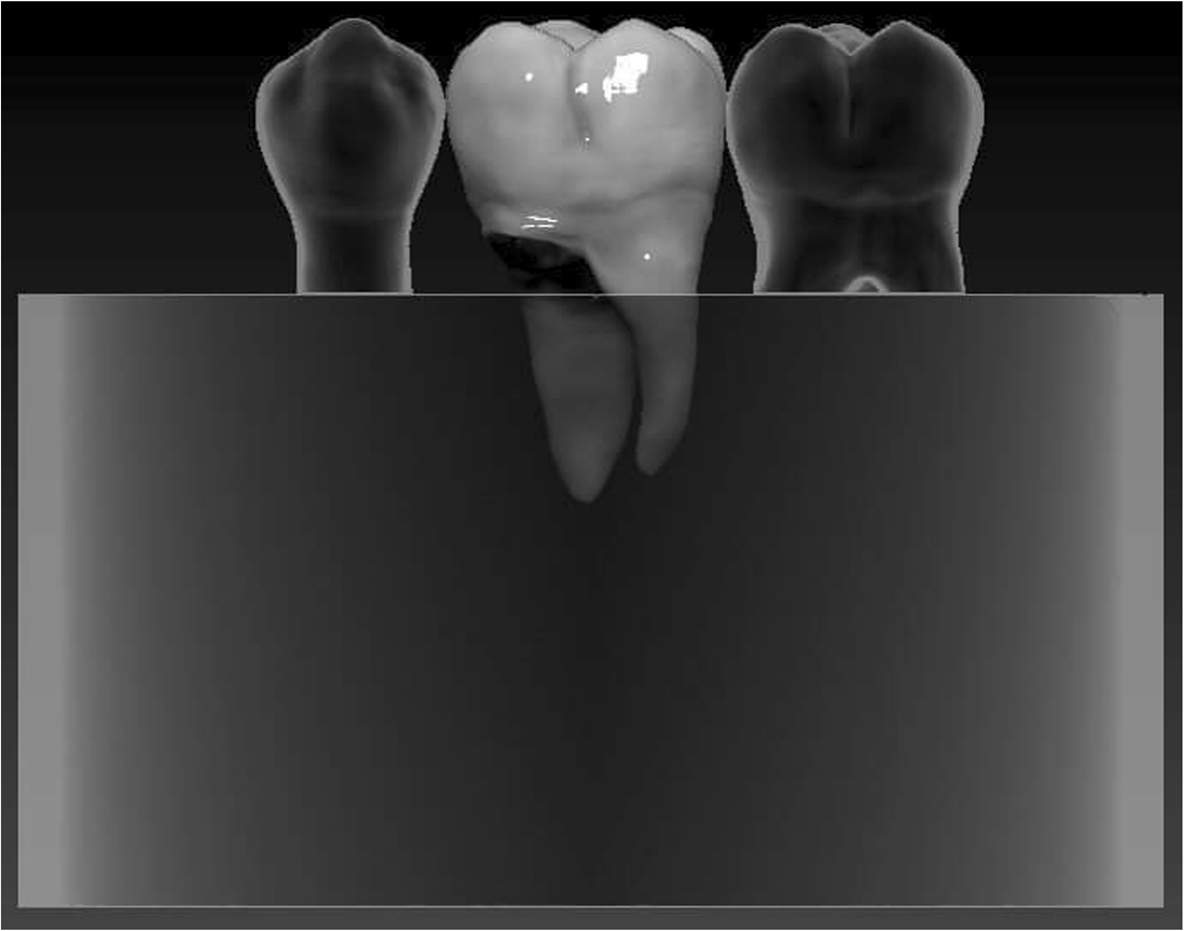
Schematic figure representing the groups (Group 2. and 4.) with a simulated Grade I. furcation involvement

The restored specimens were stored in physiological saline solution (Isotonic Saline Solution 0.9%; B. Braun, Melsungen, Germany) in an incubator (mco-18aic; Sanyo, Moriguchi, Japan) at 37 °C. Molars and premolars not selected for restoration were used as neighboring teeth to produce a tight interproximal contact on both sides. To simulate the periodontal ligament, the root surface of each tooth was coated with a layer of liquid latex separating material (Rubber-Sep, Kerr, Orange, CA) prior to embedding. Embedding was performed according to the concept published by Szabó et al. [22]. Three-teeth units were formed with the restored specimen always in the middle. Specimens in Groups 1 and 3 were embedded in methacrylate resin (Technovit 4004, Heraeus-Kulzer) at 2 mm apical from the CEJ to simulate the normal bone level (Figs. 3 and 4), while specimens in Groups 2 and 4 were embedded 3.5–4.5 mm apical from the CEJ at the level of the furcation to simulate a grade I. furcation involvement (Figs. 5 and 6).

All specimens were loaded with a crosshead speed of 2 mm/min parallel to the long axis of the tooth with a universal testing machine (5848 MicroTester1, Instron, Norwood, MA, USA) until they fractured. A 10 mm long cylindrical steel bar and 6 mm in diameter was used [23, 24]. The loading bar was positioned at the center of the occlusal surface between the buccal and palatal cusps. A force vs. distance curve was dynamically plotted for each specimen. The failure load of the specimen was defined as the load when the force versus distance graph showed an sudden drop, which can be interpreted as a decrease in the specimen’s resistance to compressive loading. Specimens were visually examined for the type and location of failure. According to Scotti and co-workers, distinction was made between restorable or non-restorable fractures (under optical microscope with a two-examiner agreement). A restorable fracture is at the level or coronally located to the CEJ, meaning that in case of fracture, the tooth can be restored, while a nonrestorable fracture extends below the CEJ and extraction is indicated [25].

Statistical analysis was conducted in SPSS 23.0 (SPSS Inc., Chicago, IL). For the comparisons between the groups, ANOVA with Tukey’s HSD post-hoc test was used. The general limit of significance was set at α = 0.05.

## Results

Table 1. summarizes the fracture thresholds for the different study groups. Groups without furcation involvement exhibited higher fracture resistance than groups with furcation involvement. Teeth restored with an indirect overlay with normal periodontal support (Group 1) yielded the highest fracture resistance (2311.6 N) among the restored groups and showed statistically significant difference compared to Group 2 (*p* = 0.038) and Group 4 (*p* = 0.011). Therefore, the null hypothesis regarding fracture resistance was rejected. There was no statistically significant difference in terms of fracture resistance between the rest of the groups. The results of the post-hoc pairwise comparisons (Tukey’s HSD) are given in Table 2.

**Table 1:** Fracture resistance values (in Newtons) and related descriptive statistics in the tested groups

Group	Valid N	Mean	Minimum	Maximum	Std. Dev.
Gr 1	15	2311.60	811.00	3858.00	894.78
Gr 2	15	1682.73	739.00	2502.00	428.64
Gr 3	15	1844.93	1059.00	3517.00	650.22
Gr 4	15	1397.33	686.00	2212.00	395.74

Groups: 1- no furcation involvement, indirect overlay; 2- furcation involvement, indirect overlay; 3- no furcation involvement, direct restoration; 4- furcation involvement, direct restoration

**Table 2:** Significance matrix from the post-hoc pairwise comparisons (Tukey’s HSD)

Group	Gr1	Gr2	Gr3	Gr4
Gr 1		***0.038596***	0.184543	***0.001153***
Gr 2	***0.038596***		0.892625	0.598182
Gr 3	0.184543	0.892625		0.215362
Gr 4	***0.001153***	0.598182	0.215362	

The conventions are the same as in Table 1. Significant differences are highlighted in bold italic numbers

In terms of the fracture patterns (Table 3), Group 3 was characterized by the highest percentage of favorable (i.e. reparable) fractures, while the rest of the groups showed dominantly unfavorable fractures. Therefore, the null hypothesis regarding fracture patterns was also rejected.

**Table 3:** Fracture patterns by group. Numbers of observations and within-group percentages. The conventions are the same as in Table 1

Fracture pattern	Gr1	Gr2	Gr3	Gr4
favorable	6 (40%)	2 (13%)	9 (60%)	5 (33%)
unfavorable	9 (60%)	13 (87%)	6 (40%)	10(67%)

## Discussion

Resective therapy has been utilized in the treatment of furcation defects for over 100 years. In our study, only maxillary first molars were root amputated and used for mechanical testing. In a retrospective study of patients with chronic or aggressive periodontal disease, maxillary molars were more frequently diagnosed with furcation lesions than mandibular molars (72% versus 50%) [26]. This was confirmed by the findings of Svärdström et al., showing that about 50% of maxillary molars in patients with varying degree of periodontal disease had at least one furcation site with deep involvement [27], whereas this number was approx. 90% in patients diagnosed for generalized advanced periodontitis [28]. In our study, different bone levels (no furcal involvement versus furcal involvement) were simulated to investigate its potential effect on fracture resistance of the tooth-restoration complex in root-amputated teeth. As pointed out by Nieri et al., the amount of bone supporting the remaining roots at the time of surgery affected the survival rate of molars with periodontal problems, and the initial bone level was found to be the most significant prognostic factor [29]. The success after root amputation has been documented by many. Park et al. found that molars with bone support > 50% around the remaining roots at the time of root resection showed a significantly higher survival rate compared to molars with < 50% bone support [11]. In our study the simulation of different bone levels seemed to have an impact on the mechanical resistance of root-amputated maxillary teeth. According to our findings, teeth with sound periodontal support (no furcation involvement, Groups 1 and 3) seemed to show a tendency to higher fracture resistance than teeth with simulated furcation involvement (Group 2 and 4). Moreover, Group 1 showed statistically significant difference in terms of fracture resistance compared to Group 2 (*p* = 0.038) and 4 (*p* = 0.011). The reason behind these findings is manifold. Partly this could be because of the impaired crown-root ratio in the periodontally compromised cases, leading to inferior results. As pointed out by Daguci et al., it is highly important that the crown–root ratio be correct to allow sufficient retention for the future restoration [30]. Also the type of coronal restaoration could have influenced the outcome (see later).

The failure rate of root-amputated molar teeth varies between 25% [11] and 38% [31, 32]. The failure is mostly mechanical (fracture), but it can also be the reoccurrence of a periapical lesion if the patient stays periodontally compromised. Ruiz et al. showed that the risk of developing apical periodontitis in endodontically treated teeth is 5.19 times higher for patients with periodontal disease compared with patients without periodontal disease [33]. To our knowledge, no one before has tested the fracture resistance of root-amputated molar teeth with simulated different bone levels. Within our study setup, the MB root of maxillary first molars was amputated. This choice had multiple reasons. Of all the canals in the maxillary first molar, the MB second canal can be the most difficult to find and treat in a clinical situation [34]. According to Degerness et al., 20% of the maxillary first molars had one canal, 79.8% two canals, and 1.1% three canals in the MB root [35]. This is in line with the findings of Cleghorn et al., showing that maxillary first molars contained a MB second canal in more than 50% of the cases in both in vivo and in vitro studies [34]. As shown by Wolcott et al., failure to detect and treat the MB second canal system will result in a decreased long-term prognosis [36], thus finding and being able to treat the MB second canal is of high importance. Nascimento et al. showed that the most frequent technical error in the endodontic treatment of maxillary molars was not obturating the MB second canal [37]. Regarding the apical part of the canals, the presence of apical delta in the MB root is much higher than those in the distobuccal or palatal root [38], which, as being difficult to clean, might compromise treatment outcome. If found and treated, since MB second canals are smaller than main canals, and often obliterated and curved [39], in retreatment cases more obstacles will arise during their negotiation. Also, the MB root has a pronounced concavity facing the internal aspect of the furcation, making access for plaque removal difficult [5]. In terms of future maintenance, in case the MB root was removed, a furcation facing anteriorly partly remains, enhancing access for interproximal brushing of the tooth [5]. Due to the above mentioned multiple reasons and based upon the clinical experience of the authors regarding possible indications for root amputation, the MB root was amputated in or study.

Regarding the coronal restorations, we tested Class I. and Class II. MOD cavities, as the literature considers these as most relevant in the case of root-amputated molar teeth. According to previous studies, Class I. cavities in root canal-treated molars can be safely restored with direct composite restorations [13, 14, 16]. Although root canal-treated teeth are weakened by the access cavity preparation process [40, 41], the presence of both marginal ridges is still protecting and “splinting” the occlusal tooth structure [16], leading to a moderate 20% reduction of cuspal stiffness [42]. Meanwhile a standardized MOD cavity preparation in maxillary premolar teeth was shown to result in an average loss of 63% in relative cuspal stiffness [43], which is related principally to the loss of marginal ridge integrity [44]. This leads to a reduction in fracture strength of approximately 54% [17, 45]. Furthermore, in root canal-treated MOD cavities the due to the extreme depth of the cavity and the lost protection of the marginal ridges, the cantilever arm increases on the remaining walls, leading to reduced fracture resistance to an extent which cannot be reinforced with a direct composite filling [17]. This is in accordance with the laboratory findings of Eapen et al. [46] and Kemaloglu et al. [47]. As stated by Seow et al., the depth of the preparation is the most critical factor in predisposing the prepared tooth to fracture [48]. Extracoronal strengthening by cuspal coverage is generally advisable [49]. Traditionally, full coverage crowns have been used, but lately adhesively placed restorations with total cuspal coverage (overlays) have been proposed as a more conservative alternative [50]. In our study, teeth restored with cuspal coverage restorations (Groups 1 and 2) showed slighty higher fracture resistance compared to the direct filling groups (Groups 3 and 4) at the same level of simulated periodontal support, though the difference was not statistically significant. The bone level together with an indirect cuspal coverage restoration seems to have a real impact on fracture resistance of root-amputated molar teeth since Group 1 was significantly stronger than teeth with impaired periodontal support (Groups 2 and 4), irrespective of their coronal restoration. Though increasing the amount of simulated periodontal support seems to increase the fracture resistance, it could not result in a significant difference when comparing the teeth restored with a direct filling (Group 3) with the simulated furcation involved groups (Group 2 and 4). Therefore, within the limitations of this study, it appears that cuspal coverage could lead to better fracture resistance values in root-amputated upper molars, clearly when accompanied with a normal bone support.

Traditionally, a considerable proportion of root canal-treated teeth would be restored in a way that involved the application of a post, in the belief that they were reinforced [7], however, as it was pointed out by several studies, fibre-reinforced composite posts fail to reinforce root canal-treated molar teeth [25, 51]. We used a short fibre-reinforced composite (SFRC) in all study groups to rebuild the missing dentine. SFRC is intended to be used in high-stress-bearing areas in both vital and non-vital teeth, especially in molars [52, 53]. As the fibers incorporated into the SFRC have a length equal to or greater than the critical fiber length (0.5–1.6 mm) [54], it can act as a stress-absorbing, dentine-replacing material [52]. In our setting, the use of SFRC in general could not shift the fracture pattern toward predominantly favorable, contrary to our previous findings [18]. It was only in Group 3 that the fracture pattern was predominantly favorable. We could only hypothesize that this might be due to the combination of the conservative direct restoration, the usage of SFRC as a core material and also a favorable bone level. In the rest of the groups, there was a shift toward unfavorable fractures. The explanation for this might be that the tested teeth were all root amputated, which not only weakens the structure, but most likely alters the stress distribution pattern as well. Group 3 also contained root amputated teeth, but in this group the simulated bone level was favorable and the coronal structure was more preserved, which could possibly account for a less devastating fracture pattern. In our study model, the treated teeth were embedded together with 1–1 neighboring tooth on both sides to form tight interproximal contacts. Creating a tight interproximal contact with the neighboring teeth or even splinting them together is important in case of root-amputated teeth. The important role of interproximal contacts was demonstrated by Krug et al. when analyzing the fracture resistance of root canal-treated premolars [55]. The authors find this a key element within the study setup, as in clinical conditions root-amputated teeth could not be left without tight contact with adjacent teeth or without splinting them to the neighbouring one.

The limitations of this investigation is that static load to fracture test was used to determine maximal fracture resistance instead of applying cyclic loading. According to Taha et al., “In experimental studies, fracture resistance to static loading has been used as a measure of the effect of cavity preparation and/or restoration on tooth strength. Although the fracture load is typically much higher than functional occlusal loads, it is still a valid method for comparing restorative materials and different cavity designs.” [56]. Also, as stated by Le Bell-Rönnlöf et al., static loading is usually the first step in the evaluation process of novel dental materials and related techniques and is commonly used in order to obtain basic knowledge regarding the fracture behavior and load capacity of a post restored tooth [57]. Notwithstanding, we would like to point out that in our opinion static loading could even be more relevant in this specific setting (i.e. root-amputated teeth with or without furcation involvement), as these teeth are more likely to fracture due to trauma or biting on a foreign object than other, non-amputated teeth. Still, this is a methodological limitation, and thus the proposed techniques need to be tested with cyclic, dynamic loading too.

## Conclusions

Within the limitations of this study, the remaining bone level after root amputation seems to have importance regarding the fracture resistance of root-amputated maxillary molar teeth restored with either direct Class I. restorations or class II. MOD indirect composite overlays with cuspal coverage.

## Supplementary information

**Additional file 1: Table S1.** Measured fracture resistance values of each specimen in the tested groups (in Newtons). Groups: 1- no furcation involvement, indirect overlay; 2- furcation involvement, indirect overlay; 3- no furcation involvement, direct restoration; 4- furcation involvement, direct restoration.

## Data Availability

The data analysed during this study are included in this article [and its additional files].

## Abbreviations

BPW: bucco-palatal width
CEJ: cemento-enamel junction
MB: mesio-buccal
MOD: mesio-occluso-distal
SFRC: short fibre-reinforced composite
TEC: traditional endodontic access
