# Supplementary material for: Fracture behavior of root-amputated teeth at different amount of periodontal support – a preliminary in vitro study
Source: BMC Oral Health. 2019 Nov 27;19:261. doi: 10.1186/s12903-019-0958-3 (PMC6882155; doi:10.1186/s12903-019-0958-3)
Supplement: Supplementary file 1 — Additional file 1: Table S1. Measured fracture resistance values of each specimen in the tested groups (in Newtons). Groups: 1- no furcation involvement, indirect overlay; 2- furcation involvement, indirect overlay; 3- no furcation involvement, direct restoration; 4- furcation involvement, direct restoration. [file 12903_2019_958_MOESM1_ESM.docx]

**Table 1.** Measured fracture resistance values of each specimen in the tested groups (in Newtons). Groups: 1- no furcation involvement, indirect overlay; 2- furcation involvement, indirect overlay; 3- no furcation involvement, direct restoration; 4- furcation involvement, direct restoration

| \| **Group 1** \| \| --- \| | \| **Group 2** \| \| --- \| | \| **Group 3** \| \| --- \| | \| **Group 4** \| \| --- \| |
| --- | --- | --- | --- | --- | --- | --- | --- |
| 1430 | 739 | 1982 | 1446 |
| 2412 | 1516 | 1905 | 1287 |
| 1179 | 1354 | 3517 | 1015 |
| 3858 | 1681 | 1675 | 1582 |
| 3752 | 1679 | 1472 | 1737 |
| 3594 | 2502 | 1059 | 1341 |
| 2361 | 1894 | 1061 | 686 |
| 811 | 1749 | 1463 | 2212 |
| 2539 | 1705 | 2104 | 1750 |
| 2705 | 1150 | 2028 | 1151 |
| 2209 | 2325 | 1329 | 1852 |
| 2049 | 1797 | 1871 | 1141 |
| 1961 | 1447 | 1550 | 1477 |
| 1770 | 1891 | 2891 | 1381 |
| 2044 | 1812 | 1767 | 902 |
